# Practice effects on the modified Concept Shifting Task (mCST): A convenient assessment for treatment effects on prefrontal cognitive function

**DOI:** 10.1186/1471-2202-12-101

**Published:** 2011-10-11

**Authors:** Karina K Kedzior, Stuti Kochhar, Hannah S Eich, Vikram Rajput, Mathew T Martin-Iverson

**Affiliations:** 1 School of Humanities and Social Sciences, Jacobs University Bremen, Campus Ring 1, 28759 Bremen, Germany; 2 Clinical Neurophysiology Unit, Graylands Hospital, John XXIII Avenue, Mt Claremont 6010, Perth, Australia; 3 Pharmacology & Anaesthesiology Unit, School of Medicine & Pharmacology, Faculty of Medicine, Dentistry & Health Sciences, The University of Western Australia, 35 Stirling Highway, Crawley 6009, Perth, Australia

**Keywords:** modified Concept Shifting Task (mCST), Trail-Making test, practice effects, meta-analysis, prefrontal cognitive function

## Abstract

**Background:**

Trail-making tests, such as the Concept Shifting Task (CST), can be used to test the effects of treatment on cognitive performance over time in various neuropsychological disorders. However, cognitive performance in such experimental designs might improve as a result of the practice obtained during repeated testing rather than the treatment itself. The current study investigated if practice affects the accuracy and duration of performance on the repeatedly administered Concept Shifting Task modified to make it resistant to practice (mCST). The mCST was administered to 54 healthy participants twice a day, before and after a short break, for eight days. Results. The ANOVA and meta-analysis showed that there was no improvement in the mCST accuracy on the last vs. the first trial (Hedges' *g *= .14, *p *= .221) or within the session (after vs. before the break on all days; *g *= .01, *p *= .922). However, the participants performed the task faster on the last vs. the first trial (*g *= -.75, *p *< .001) and after vs. before the break on all days (*g *= -.12, *p *= .002). Conclusions. Repeated administration of the mCST does not affect the accuracy of performance on the test. However, practice might contribute to faster performance on the mCST over time and within each session.

## Background

Trail-making tests, such as the Concept Shifting Test (CST) [1], can be used to investigate higher cognitive processes, including the ability to shift attention and the strategy to perform the test. The CST is a simple pen-and-paper test which requires the participants to cross out, as fast as possible, a set of either empty circles (a control trial), letters, numbers, or a combination of both (thus requiring concept shifting between two stimuli types) in alphabetical or numerical order [1]. The cognitive processes used to perform the task include attention, visual recognition, long-term memory, and visual scanning [1]. Among others, the test has been used in clinical practice to investigate cognitive performance in schizophrenia [2] and depression [3].

Similar to other classic tests of neurocognitive function, such as the Wisconsin Card Sorting Test or the Wechsler Memory Scale- Revised, the CST is likely to suffer from practice effects when administered repeatedly [4-6]. "Practice effects are defined as increase in a subject's test score from one administration to the next in the absence of any interventions" [[4]/p. 1]. Thus, even though the CST is useful in clinical practice and research because it is easy to administer and score, inexpensive, and brief compared to the other classic neurocognitive tests, the improvement of performance on the CST might not necessary indicate an improvement in cognitive functioning but result from practice. Therefore, a modified version of the CST (mCST) was developed by the current authors (V. R. and M. M-I.) to make the task less predictable (harder to learn) and thus presumably more robust against potential practice effects. Specifically, the CST was modified to increase the number of trials performed per session (from four in the CST to eight in the mCST). Similar to the CST, on the mCST participants are required to cross out, as fast as possible, a set of either letters or numbers either in ascending (alphabetical or numerical) or descending (reverse alphabetical or from highest to lowest) order. However, in contrast to the CST, such a task instruction is reversed half way through the eight trials (on trial five) on the mCST. Therefore, a concept shift on the mCST includes a shift in stimulus type (either letters or numbers) from trial to trial and, in addition, a shift in the strategy needed to complete the task (following the ascending or the descending instruction).

The mCST paradigm is very similar to the so-called task-switching paradigms [7,8] which are used to study participants' mental flexibility when switching from one task to another (for example, switching between addition and multiplication). Switching between two tasks induces slower responses (longer reaction-times, RT) with greater error rates on the second task compared to repeating the first task. Interestingly, such switch-costs are robust to prolonged practice although the mechanism behind this robustness is unclear [8]. In general, the presence of switch-costs can be explained, among others, using two competing theoretical approaches. The reconfiguration approach suggests that switch-costs reflect the time needed to endogenously reconfigure the task-set for the new task, where the task-set is the "collection of control settings or task parameters that program the system to perform processes such as stimulus identification, response selection, and response execution" [[8]/p. 601]. In contrast, the interference approach suggests that the switch-costs might arise from the exogenous interference of the earlier task with the present one [8].

While changes in error rates (expressed as either % error or % accurate responses) and RTs can be used to assess the impact of practice in task-switching paradigms, the RTs were not measured on the mCST. Specifically, each trial on the mCST consists of multiple stimuli appearing simultaneously (10 letters or numbers). While the RT to each individual stimulus on each trial was not of interest, the effects of practice can be investigated by measuring both the accuracy of performance (% accurate responses) and the change in the total duration of performance (between crossing out the first and the last stimuli) on each mCST trial.

The aim of the current study was to test for practice effects on the mCST administered to healthy participants. The task was administered in five separate studies (unpublished to date) using different methods (where the participant groups, form of task administration and scoring methods differed; for more detail refer to the Methods section). The specific aim of all five studies was to test if the error rates (expressed as % accurate responses) and duration of performance (time, in s, between selecting the first and the last stimulus on each trial) on the mCST would change over time and within each testing session. Specifically, the mCST was administered for eight days, twice a day (before and after a 30 min break). In clinical settings, such a design would allow to test performance on the mCST before and after some treatment (acute effects of treatment) and before the first and after the last treatment (chronic effects of treatment). An additional aim of the study was to investigate if the results depend on the mode of administration of the mCST (paper vs. electronic).

The main hypothesis of the study was that if practice does affect the task then performance on the mCST would differ or be better in the short-term (after vs. before the break on each day) and/or in the long-term (on the last vs. the first trial). Furthermore, if the performance on the task depends on the study characteristics (methodology, participants) then the results obtained in each of the five methodologically-heterogenous studies would differ and such differences would require further investigation. Finally, it was hypothesised that if performance on the mCST depends on the mode of administration (computerised vs. paper) then a different pattern of results would be noted if the five studies were grouped into two, depending on the mode of task administration.

## Methods

### Participants

All research described in the manuscript conformed to the ethical guidelines recommended by the Declaration of Helsinki, and was approved by the Research Ethics Committees at the University of Western Australia and North Metropolitan Health Services in Perth, Australia. Following a written informed consent a total of 54 participants took part in five separate studies conducted in Australia and Germany, between 2005-2010 (see Table 1 for study details). All participants were task-naïve and were selected from either the undergraduate students at Jacobs University Bremen (Germany) or the staff and students at the North Metropolitan Health Services (Australia).

**Table 1:** The methodological details of the five studies reported in the current article.

Study no.Country Year	*N* *Males* *% male*	Participants Age *M ± SD*	Task administration mode	Randomisation of trials	Task instruction	Data scoring method
1 Australia 2005	8 6M 75%	Students/ Staff 39 ± 15	Paper	Pseudo-random	verbal	Real time- stop watch/video-recording

2 Germany 2009	15 9M 60%	Students 21 ± 1	Paper	Pseudo-random	verbal/written	Real time- stop watch

3 Germany 2009	9 3M 33%	Students 21 ± 3	Paper	Pseudo-random	verbal/written	Real time- stop watch

4 Germany 2010	16 5M 31%	Students 20 ± 1	Electronic	Fully-random	written	Computerised

5 Germany 2010	6 1M 17%	Students 20 ± 1	Electronic	Fully-random	written	Computerised

Note. All data collection was done in the English language (in Germany the study was conducted at an English-speaking Jacobs University Bremen).

### Task

**Figure 1:**
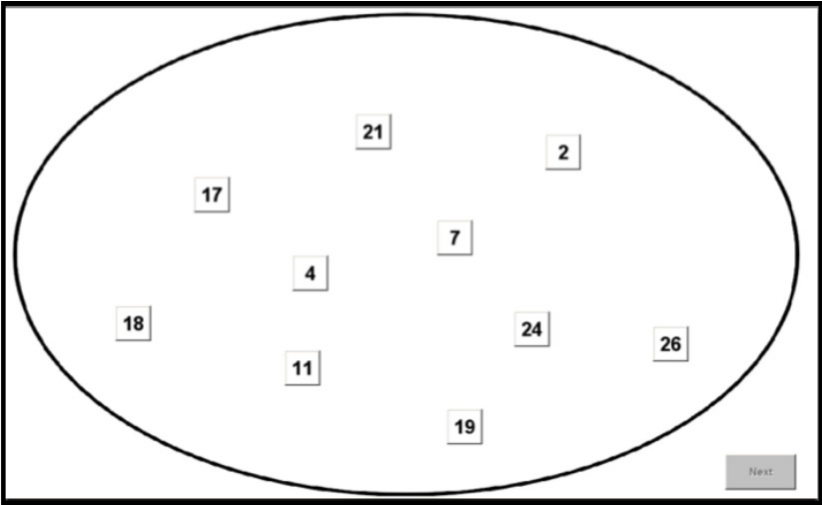
**An example number trial on the computerised mCST**. Having clicked on all numbers in either ascending (numerical) or descending (reverse numerical) order (depending on the instruction presented to the participant on the screen before trial 1) the participant had to click on the Next button to move on to the next trial (in this case, letters). Following trial 4 a new instruction would be shown on the screen (to complete the task in the opposite order to the one on trial 1-4) and subsequently trials 5-8 would be shown.

The mCST was administered either on paper or electronically. The paper task consisted of eight trials during which each participant was instructed to cross out, as fast as possible, 10 numbers or 10 letters randomly selected from a set of 26 numbers (1-26) or 26 letters (A-Z) respectively, either in ascending (numerical or alphabetical) or descending order (see Figure 1 for a sample electronic trial). The paper task was pseudo-randomised in that the set of numbers or letters alternated among all trials of the experiment (for example, if trial 1 consisted of letters then trial 2 consisted of numbers followed by letters and so on), while the order of number and letter sets always remained the same for every participant. The task instruction remained constant on trials 1-4 and was then reversed before trial 5 and remained constant on trials 5-8. Therefore, the task incorporated both concept shifts and attention shifts by changing the rules used to respond to the same set of stimuli, in one case altering which type of stimulus is relevant (letter or number), and in the other case changing the instruction used (ascending or descending).

The duration of performance was the time to complete each trial on the task (in seconds) and the accuracy of performance was the number of correctly crossed out numbers or letters (out of 10 per trial) expressed as percentage score. The duration and accuracy of performance were recorded on trials 1, 5 and 8. The participants were not required to correct their errors while completing the task. The error in accuracy of performance was recorded if a participant started with an incorrect item, skipped an item, or ignored the instruction. For example, if G was crossed out before C on a letter trial to be completed in ascending order and all other letters were crossed out correctly then the accuracy on this trial was 9/10 or 90%.

The electronic version of the mCST, written in MatLab 2009 (MathWorks, USA), was identical to the paper mCST described above with two exceptions. First, on the computerised mCST the trials were presented on a computer screen and the participants were required to click, as fast as possible, on letters or numbers which would subsequently become unavailable to be clicked on again to mimic the crossing of letters or numbers on paper. Secondly, in contrast to the paper version, the electronic mCST was fully randomised in that the set of numbers or letters randomly alternated among all trials and all participants.

There was no practice trial. All 10 stimuli (letters or numbers) were presented simultaneously in each trial in both the paper and the electronic versions of the task (Figure 1).

### Procedure

The participants completed the mCST before and after a 30 min break for eight days. The participants were free to do what they wished during the break. In case they missed any of the experimental days the performance on their first and last day was taken into account when computing performance duration and accuracy.

## Results

### Accuracy and duration of performance of all participants on all eight days

**Figure 2:**
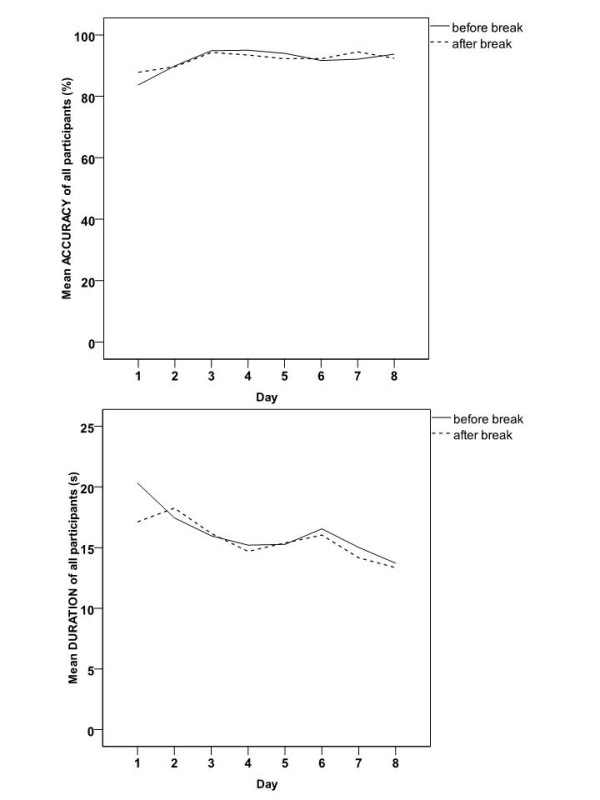
**The mean accuracy and duration of performance of all participants in all five studies, on the mCST on each of the eight experimental days before and after the session break**.

The mean accuracy and duration of performance of all participants in all five studies is shown in Figure 2. In general, the accuracy did not improve on the last vs. the first mCST trial and the mean accuracy did not reach the ceiling of 100%. On the other hand, the duration of performance improved (was reduced) on the last vs. the first mCST trial. Despite the difference in improvement between the two measures, there was a strong negative correlation between the two variables (as duration decreased, accuracy increased); Pearson's *r *= -.81, *p *(two-tailed)<0.0005, *N *= 16.

### Performance on last vs. first mCST and after vs. before break in five studies

To investigate if performance on the mCST shown in Figure 2 differed significantly, four repeated measures ANOVAs were conducted in SPSS18, each with one between-subject factor, STUDY, with 5 levels (corresponding to the five studies that had used the mCST) and the following within subject factors:

1. Dependent variable: ACCURACY, one within-subject factor TRIAL with 2 levels (last vs. first mCST trial)

2. Dependent variable: DURATION, one within-subject factor TRIAL with 2 levels (last vs. first mCST trial)

3. Dependent variable: ACCURACY, one within-subject factor BREAK with 2 levels (after vs. before break on all days)

4. Dependent variable: DURATION, one within-subject factor BREAK with 2 levels (after vs. before break on all days).

Any significant main effects and/or interactions were investigated using single comparisons with Sidak's correction.

The accuracy of performance did not significantly change between either the last vs. the first mCST trial, after vs. before the break, or among the five studies (Table 2). Both interactions, TRIAL×STUDY and BREAK×STUDY on the accuracy of performance, were also non-significant (Table 2).

**Table 2:** The results of four repeated measures ANOVAs comparing either the accuracy or the duration of performance on either the last vs. the first mCST trial or after vs. before the session break on all experimental days.

Effects	*df; df_error_*	*F*	*p_two-tailed_*	*η^2^_part_*	power
**last vs. first mCST TRIAL**					
**ACCURACY**					
TRIAL	1; 49	1.97	.167	.04	.28
STUDY	4; 49	.71	.587	.06	.21
TRIAL×STUDY	4; 49	.42	.791	.03	.14
**DURATION**					
TRIAL	1; 49	12.45	.001*	**.20**	.93
STUDY	4; 49	1.91	.124	.14	.54
TRIAL×STUDY	4; 49	.65	.628	.05	.20

**after vs. before BREAK (all days)**					
**ACCURACY**					
BREAK	1; 49	.31	.581	.01	.09
STUDY	4; 49	1.14	.350	.09	.33
BREAK×STUDY	4; 49	.38	.826	.03	.13
**DURATION**					
BREAK	1; 49	6.82	.012*	**.12**	.73
STUDY	4; 49	1.35	.266	.10	.39
BREAK×STUDY	4; 49	.63	.646	.05	.19

Note. *df*- degrees of freedom, *η^2^_part_*- partial eta squared (measure of effect size).

*p < .05

The analyses of duration of performance revealed two significant main effects of TRIAL and BREAK (Table 2). These effects were due to participants performing significantly faster on the last (*M ± SEM *15.74 ± 0.76 s) vs. the first (*M ± SEM *19.93 ± 1.10 s) mCST trial and after (*M ± SEM *15.73 ± 0.58 s) vs. before (*M ± SEM *16.27 ± 0.54 s) the session break. Both interactions and the main effect of STUDY on the duration of performance were non-significant (Table 2). Thus, it appears that the duration of performance on the mCST improved at the end vs. the beginning of the study and after vs. before the session break on all days. Among others, practice might be one of the explanations for such an improvement.

The interpretation of the non-significant results in the current study is difficult due to the low power associated with all the results listed above (range: .09-.54 in Table 2) and thus high chances of having committed Type II error or simply having 'missed the opportunity' to detect significant differences among groups. The modern approach to psychological data analysis is that of focusing on the effect sizes and associated confidence intervals rather than significance testing using the arbitrary threshold of 0.05 as the level of statistical and meaningful significance of results [9,10]. In accordance with this approach, and considering that the data pooling done in the ANOVA is not fully justifiable here due to differences among the studies listed in Table 1 (even if these differences were not detected using the standard significance testing approach shown in Table 2), the effect sizes of all studies were investigated by combining them together using meta-analysis.

Meta-analysis is a way of statistically summarising the results of a number of studies following a systematic literature review of published and unpublished scientific work [10]. Meta-analysis can also be used to summarise the results of studies that were collected using different methods and/or from different samples, such as in the present case (Table 1). Unlike the standard statistical procedures that heavily rely on testing for presence or absence of statistical significance, meta-analysis focuses on effect sizes that can be computed from individual studies and then mathematically averaged to obtain one effect size describing the degree of association in the combined studies. In contrast to computing an arithmetic mean of all the individual study effect sizes, the meta-analytical mean is weighted such that more precise studies have a higher contribution than the less precise studies to the mean weighted effect size of all the studies [10].

The current hypotheses were rephrased for the purpose of meta-analysis. Specifically, it was hypothesised that if the mCST is affected by practice then the effect sizes should be high (indicating high differences in means) and/or in the expected directions (positive for accuracy and negative for duration) in the short-term (after vs. before the break on each day) and the long-term (on the last vs. the first trial). On the other hand, the presence of low effect sizes (small differences in means) in the directions opposite to those expected (negative for accuracy and positive for duration) would indicate that practice did not improve the performance on the mCST.

The results of the analysis of duration and accuracy of performance for each study were entered separately into Comprehensive Meta-Analysis 2.0 (CMA; Biostat, USA). A meta-analysis using the random-effects model according to Hedges' et al. method [10] was performed separately for duration and accuracy of performance to compare the effect sizes between the last vs. the first mCST trial or after vs. before session break on all days in agreement with the comparisons reported in the ANOVA above (Table 2).

The mathematical details of the random-effects meta-analysis are described elsewhere [10]. Briefly, based on the data collected in each individual study (*N*, *M ± SD *of performance before and after, and Pearson Product Moment correlation coefficient *r *between performance before and after) the CMA computes a chosen effect size for each study (Hedges' *g *in the current study) and its variance. The Hedges' *g *is a corrected for the sample size version of a commonly used effect size measure, Cohen's *d *(a standardised mean difference). The Cohen's *d *is often too large in studies utilising small samples (as was the case in all five studies here), and thus using *g *prevents the overestimation of the overall effect size in small-*N *studies [10]. In the current study the use of both effect sizes, *g *and *d*, produced the same results. For interpretation purposes Cohen's *d *is reported in the Results section together with Hedges' *g*. The interpretation criteria for the absolute size of Cohen's *d *are: *d *= .20-.49 (small), *d *= .50-.79 (moderate), and *d*≥.80 (large) [11].

The random-effects meta-analysis works by assigning a weight to each study's effect size and combining such weighted effect sizes into an overall mean weighted effect size for all studies. The weight in random-effects meta-analysis is the inverse of the sum of the within- and between-study variance. The mean weighted effect size is a sum of products of the weight and effect size in each study divided by the sum of all weights in all studies. CMA also computes the precision of the mean weighted effect size (variance and the lower and upper 95% confidence intervals, *95%CI*) and a *z*-score to test if the mean weighted effect size is significantly different from zero (meaning no difference in performance between the last vs. the first mCST trial and after vs. before the break on all days). It needs to be noted that such a significance testing of the mean weighted effect size is also prone to the same problems as those stated above for the ANOVA (Type I and II errors).

In addition, the heterogeneity among the studies in meta-analysis is tested using a *Q*-statistic and an *I^2 ^*index [10]. The *Q*-statistic tests the null-hypothesis that there is no heterogeneity in effect sizes among the studies included in the analysis. The *I^2 ^*index expresses the *Q*-statistic on a 0-100% scale and can be interpreted as the amount of variability among studies due to real differences among studies (as opposed to differences due to chance alone).

The random-effects model was chosen because of the assumption that there would be between-study heterogeneity due to the differing methods utilised by the five studies (Table 1). The mean weighted effect size computed using the random-effects method is not overly influenced by any extreme studies if the between-study variance is non-zero. Even if studies appear to be homogenous (*Q*-statistic not significantly different from zero), the random-effects model allows for inferences about the wider population compared to the other method of meta-analysis, the fixed-effect model, that only provides a descriptive analysis of the included studies without extrapolating to the population [10].

**Figure 3:**
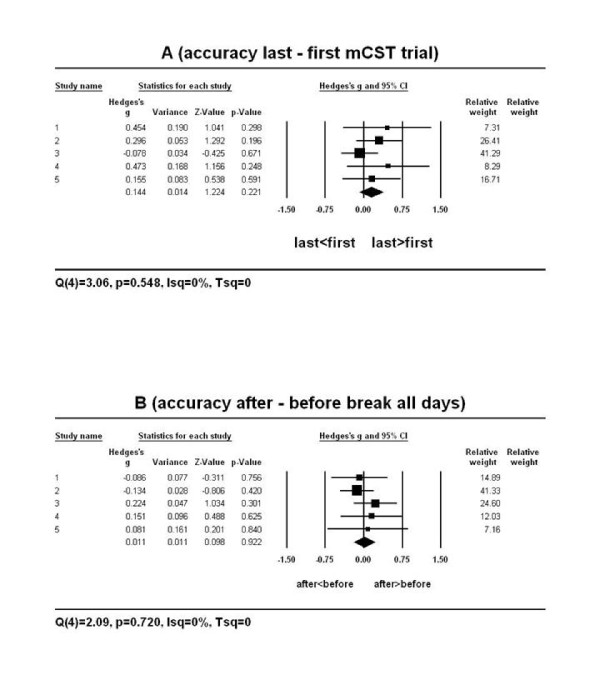
**The forest plot of the random-effects meta-analysis on the mean ACCURACY of performance on the last vs. the first mCST trial (A) and after vs. before the session break on all experimental days (B)**. Positive effect sizes (Hedges' *g *represented as boxes on the plot) indicate an improvement while negative effect sizes indicate a decline in accuracy of performance on the last vs. the first mCST trial or after vs. before the break. The *95%C.I*. of all five effect sizes (the horizontal lines through the boxes) overlapped with zero. The mean weighted effect sizes *g *(the centre of each diamond in A and B) were small and their *95%C.I*. (the edges of the diamonds) overlapped with zero. Therefore, there was no change in the mean accuracy of performance in the long-term (last vs. first mCST trial; A) or the short-term (after vs. before the session break; B). The relative weights indicate that the study 3 in A and the study 2 in B had the highest contribution to the computation of the mean weighted effect sizes.

In the current study the results of meta-analysis confirmed the results of the ANOVAs for both, the accuracy and the duration of performance on the mCST. Specifically, there was no difference between the mean accuracy of performance on the last vs. the first mCST trial in all five primary studies (Figure 3A). A mean weighted effect size of all five studies together was small and its confidence interval included zero (Hedges' *g *= .14, 95%*CI: *-.09 to .38 or Cohen's *d *= .24, 95%*CI: *-.03 to .51). Similarly, the mean accuracy did not differ after vs. before the session break on all experimental days in all five primary studies (Figure 3B). A mean weighted effect size of all five studies together was small and its confidence interval included zero (*g *= .01, 95%*CI: *-.20 to .22; *d *= .03, 95%*CI: *-.24 to .30). Therefore, it appears that practice was unable to improve participants' accuracy on the mCST despite it being completed on average 16 times a day for eight days.

**Figure 4:**
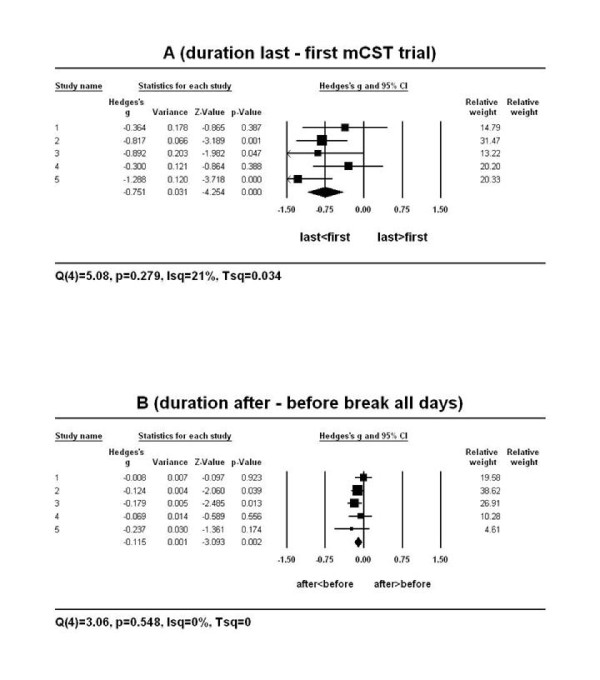
**The forest plot of the random-effects meta-analysis on the mean DURATION of performance on the last vs. the first mCST trial (A) and after vs. before the session break on all experimental days (B)**. Positive effect sizes (Hedges' *g *represented as boxes on the plot) indicate a slower while negative effect sizes indicate a faster duration of performance on the last vs. the first mCST trial or after vs. before the break. The *95%C.I*. of some effect sizes (the horizontal lines through the boxes) overlapped with zero. The mean weighted effect sizes *g *(the centre of each diamond in A and B) were medium (A) and small (B) and their *95%C.I*. (the edges of the diamonds) did not overlap with zero. Therefore, the mean overall effect sizes show that participants performed the task faster in the long-term (last vs. first mCST trial; A) and short-term (after vs. before the break within each session; B). The relative weights indicate that the study 2 in A and B had the highest contribution to the computation of the mean weighted effect sizes.

In contrast to the accuracy of performance and again in agreement with the ANOVA, some of the primary studies and the mean weighted effect size were different from zero when comparing the duration of performance on the last vs. the first mCST trial (Figure 4A) and after vs. before the session break on all experimental days (Figure 4B). The mean weighted effect sizes were low-moderate and their confidence intervals did not overlap with zero (last vs. first mCST: *g *= -.75, 95%*CI: *-1.10 to -.41 or *d *= -.70, 95%*CI: *-1.19 to -.21; after vs. before session break: *g *= -.12, 95%*CI: *-.19 to -.04; *d *= -.37, 95%*CI: *-.65 to -.09). The negative sign of the mean weighted effect sizes indicates that the duration of performance improved on the last vs. the first mCST trial and after vs. before the break on all days. Such an improvement in duration of performance on the mCST could be attributable to the effects of practice.

### Performance on paper vs. electronic mCST

To investigate if the results of the study differed depending on the mode of administration of the mCST task, all participants were divided into two groups based on the type of task they completed- paper (*N *= 32) vs. electronic mCST (*N *= 22). The dependent variables were either the mean accuracy or the mean duration of performance on the mCST each computed from all trials before and after the break on all days. Two independent samples t-tests showed that both the mean accuracy and the mean duration of performance on the mCST did not differ significantly between the paper and the electronic versions of the task (Table 3). The effect sizes for the difference in performance between the two modes of the mCST administration were small for both the accuracy and the duration of performance.

**Table 3:** The results of independent samples t-tests comparing either the mean accuracy or the mean duration of performance between the paper and the electronic mCST tasks.

Dependent variable	*M ± SEM*		*t (df)*	*p_two-tailed_*	*d*
	*Paper N = 32*	*Electronic N = 22*			
**ACCURACY**	92.65 ± .99	90.48 ± 1.14	1.43 (52)	.160	.40
**DURATION**	15.43 ± .71	16.98 ± .71	-1.48 (52)	.144	-.41

Note. *df*- degrees of freedom, *d*- standardised difference in means (measure of effect size).

## Discussion

The results of the current study suggest that the accuracy of performance on the modified Concept Shifting Task (mCST) is robust to the effects of practice that could arise from a repeated completion of this test over the average of eight days, twice a day (before and after a short break). Specifically, the effect sizes (both Hedges' *g *and Cohen's *d*) were small (all <.24) when comparing the accuracy on the last vs. first test performance and after vs. before the session break on all experimental days. Therefore, the accuracy on the mCST is a useful measure for monitoring the frontal-cortical cognitive functioning over time.

On the other hand, practice appeared to contribute to faster completion of the test at the end compared to the beginning of the experiment. The resulting effect sizes of this comparison were moderate (absolute values of *g *and *d *of .75 and .70 respectively). Similarly, the participants were faster after vs. before the session break on all testing days but the effect sizes of this comparison were small (absolute values of both *g *and *d *< .37). Therefore, even though the accuracy of performance on the mCST was robust against effects of practice, the duration of performance improved with time, especially in the long-term (on the last vs. the first mCST trial). Since studies of practice effects typically focus on task accuracy only [for example see [4]], the effects of practice on the time to complete a test remain relatively unreported. However, this might be of clinical relevance. For instance, depression appears to be associated with a reduced speed of information processing [3] and thus it would be expected that depression patients would be slower at completing the task than healthy controls. The current results suggest that it might be difficult to evaluate an improvement in the speed of performance on the mCST following a certain treatment in depression because such an improvement could be confounded by practice effects.

The improvement in the duration of performance on the mCST is likely to be due to the effect of practice and learning in that participants were familiar with the general framework of the experiment, including the instruction change half way through each experimental session and the type of stimuli used (letters or numbers). In general, the presence of practice effects in serial testing is associated with activation of various cognitive mechanisms, such as executive functions and memory [4]. Both, short-term and procedural memory are likely to be associated with the short-and the long-term improvements in duration on the mCST based on the finding that practice effects are consistently observed on neurocognitive tests of memory [4-6]. Consequently, the absence of practice effects on the duration of performance on the mCST could indicate potential deficits in cognitive functioning [6] and thus deem the test suitable for clinical applications.

Another reason for the improvement in the duration of performance on the mCST could be a shift in the bias of the participants' trade-off between accuracy and speed of performance. Specifically, at the beginning of the experiment, participants may be more biased towards accuracy, whereas after multiple testing, the bias may be shifted towards completing the task faster. However, against this argument is the finding that the higher accuracy was significantly correlated with the faster performance (lower duration) in the current study (Figure 2) suggesting that participants were either fast and accurate or slow and inaccurate on the mCST. The problems with changing trade-offs between accuracy and speed in psychological tests may occur less frequently in clinical samples, where the tests may be perceived as being more relevant to own health of the participants. Specifically, participants in clinical settings may maintain motivation to engage with the task, as it has direct and personal relevancy. Thus, if the change in speed of performance is due to trade-offs in motivation, it is not clear if the findings of an improvement in speed observed in healthy controls would generalise to patient samples. However, if the speed improvement is due to practice effects, then patients should also show an increase in speed over time regardless of their motivation to complete the task.

In task-switching paradigms, task-switching leads to slowing-down (increasing) of RTs [7,8]. Since the RTs were not measured in the current study it can only be speculated that despite the overall decrease in the duration of performance over time, the RTs (the time taken to cross out the first letter or number) might have increased due to the participants either needing to endogenously reconfigure the task set or experience the exogenous interference from earlier instructions.

Therefore, a decrease of duration on the mCST might have been indirectly due to both exogenous and endogenous processes affecting the RTs to the task stimuli in the current study. In other words, it can be speculated that participants might have taken longer to execute their first response (for instance, identify the letter or the number to be crossed out first on each trial) but afterwards completed the trials faster. Alternatively, the current participants completed the mCST faster with time because they could reconfigure the task set quicker and/or because there was less interference from new instructions presumably from learning the procedural aspects of the task towards the end of experiment. Given that it was possible for participants to predict the next instruction (crossing of letters or numbers in either ascending or descending order) but not the composition of the next trial (which letters or numbers would be presented and in what precise position) offers an explanation for why improvement was found only for duration but not for accuracy of performance on the mCST.

The lack of practice effects on the accuracy of performance in the current study might be due to employing test forms (or computer screens) with varying types and locations of stimuli. It has been shown that, compared to identical test forms, alternate forms reduce practice effects on some memory tests [12]. Furthermore, practice effects are also moderated by task difficulty [5]. It appears that the current task was difficult to learn, since it was impossible to memorise the precise location and type of stimuli on each trial, which changed with each trial. Therefore, even though it may not be possible to completely remove the effects of practice from serial psychological testing, the current mCST task appears to be resistant to practice in terms of performance accuracy in healthy participants. Therefore, the task might be of use to assess treatment effects over time in clinical samples.

One of the limitations of the current study is the method of meta-analysis used. As stated in the Results section, the current study used the random-effects model due to the assumption that the five studies used in the analysis differed from each other methodologically and thus would most likely not share one common effect size. However, when the number of studies used in meta-analysis is low then the estimation of the between-study variance is compromised [10]. In this case, the results of meta-analysis should not be generalised to a wider population. One way of dealing with this problem would be to conclude that the mean weighted effect size describes the studies in the current analysis only or even to refrain from computing a mean weighted effect size at all [10]. This solution appears problematic for at least two reasons. Firstly, there is no consensus on what constitutes a 'small' number of studies in meta-analysis. Typically, in clinical research, a large number of studies located for a purpose of meta-analysis (for instance, *N*>2000) is drastically reduced to *N *< 10 mainly due to the inability to extract adequate information from such sources. Thus, it is not uncommon to perform meta-analysis on as few as five primary studies [for example, see [13]]. Secondly, even though not reported here, the results of the fixed-effect meta-analysis conducted on the current five studies produced the same results as the random-effects model in terms of similar mean weighted effect sizes, *95%CI*s and *p *-values. Furthermore, the effect sizes in the five primary studies were similar to each other and to the mean weighted effect size and the results of meta-analysis were in agreement with the classical statistical analysis (ANOVA) performed on the data. Therefore, regardless of the method of meta-analysis, it appears that the mean weighted effect sizes accurately describe at least the current primary studies, especially for the accuracy data (Figure 3A and 3B).

Even though it contains a small number of primary studies, the strength of the current study is that all the studies employing the mCST to date were included in the analysis and that primary data from all five studies were available to the authors, which is uncommon in a typical meta-analysis. Therefore, the results of the current meta-analysis are not affected by a publication bias in terms of not including all available studies on the topic due to a limited search strategy, biasing the search for primary studies to the English language only (the Tower of Babel Error), and not including non-significant primary results which may not have been published (the File-Drawer Problem) [10].

Another limitation of the current work is that it was impossible to investigate practice effects on the mCST in more detail due to the limited amount of data available on this new task. For instance, other studies utilising meta-analysis of practice effects in other psychological tasks compared subgroups of studies or performed moderator analyses to find out what factors might contribute to practice effects, particularly if heterogeneity among studies was detected [14,15]. In general, factors such as age, gender, and education were found to affect the performance on the original CST task [1] and thus the mCST should also be administered to larger samples to test for factors other than practice that can affect performance on this task.

Practice effects in psychological tests also depend on the number of repetitions of the task and the temporal proximity of repetitions [4]. The five primary studies analysed in this article utilised the mCST for the average of eight days only which might have been too short for the effects of practice to occur. On the other hand, this relatively short administration period was adequate for the participants to show practice-related improvement in duration of performance. It would be of interest to test the effects of practice on this task over a longer period of time, such as one year. So far, preliminary evidence from Study 1 that continued on for 20 days suggests that accuracy of performance did not improve over time (the last vs. the first mCST trials) while participants completed the task faster over time (unpublished data). Therefore, preliminary data support the overall results collected over eight days and suggest that the mCST is prone to practice effects in terms of duration of performance but not the accuracy of performance on up to 20 days of testing. An experimental design utilising the mCST over a number of months would be better comparable to clinical protocols which may require patients to complete the mCST over a longer period of time and less frequently than daily to investigate the long-term effectiveness of some treatment.

## Conclusions

In conclusion, the accuracy of performance on a simple modified Concept Shifting Task (mCST) appears to be robust to the effects of practice in healthy participants following multiple administration of the task for up to eight days. Therefore, the task might be suitable for testing cognitive deficits in clinical research. Caution should be applied when evaluating the duration of performance on the mCST since it appears that the task is performed faster with time, most likely due to practice. The effects of practice on the mCST administered for more than eight days remain to be tested.

## Abbreviations

mCST: modified Concept Shifting Task.

## Authors' contributions

All authors read and approved the final manuscript; KK wrote the manuscript, performed all analyses and supervised data collection in studies 2-5; SK and HE collected data in studies 2-5, performed the primary analyses and preliminary meta-analyses; VR and MMI designed the mCST and collected and analysed data in study 1.
